# Visible-Light Hyperspectral Reconstruction and PCA-Based Feature Extraction for Malignant Pleural Effusion Cytology

**DOI:** 10.3390/bios15110714

**Published:** 2025-10-28

**Authors:** Chun-Liang Lai, Kun-Hua Lee, Hong-Thai Nguyen, Arvind Mukundan, Riya Karmakar, Tsung-Hsien Chen, Wen-Shou Lin, Hsiang-Chen Wang

**Affiliations:** 1 Division of Pulmonology and Critical Care, Department of Internal Medicine, Dalin Tzu Chi Hospital, Buddhist Tzu Chi Medical Foundation, No. 2, Minsheng Road, Dalin, Chiayi 62247, Taiwan; laicl.dalin@gmail.com; 2 School of Medicine, Tzu Chi University, 701 Zhongyang Rd., Sec. 3, Hualien 97004, Taiwan; 3 Department of Trauma, Changhua Christian Hospital, No. 135, Nanxiao St., Changhua City 50006, Taiwan; 88847@cch.org.tw; 4 Department of Mechanical Engineering, National Chung Cheng University, 168, University Rd., Min Hsiung, Chiayi 62102, Taiwan; hongthai.nguyen@tnut.edu.vn (H.-T.N.); arvindmukund96@gmail.com (A.M.); karmakarriya345@gmail.com (R.K.); 5 Department of Mechanical Engineering, Thai Nguyen University of Technology, No. 666, Street 3/2, Thai Nguyen City 250000, Vietnam; 6 Department of Biomedical Imaging, Chennai Institute of Technology, Sarathy Nagar, Chennai 600069, Tamil Nadu, India; 7 School of Engineering and Technology, Sanjivani University, Sanjivani Factory, Singnapur, Kopargaon 423603, Maharashtra, India; 8 Department of Internal Medicine, Ditmanson Medical Foundation, Chia-Yi Christian Hospital, Chiayi 60002, Taiwan; cych13794@gmail.com; 9 Neurology Division, Department of Internal Medicine, Kaohsiung Armed Forces General Hospital, 2, Zhongzheng 1st.Rd., Lingya District, Kaohsiung City 80284, Taiwan; 10 Technology Development, Hitspectra Intelligent Technology Co., Ltd., Kaohsiung 80661, Taiwan

**Keywords:** malignant pleural effusion, hyperspectral imaging, computer-aided diagnosis, principal component analysis, spectroscopy

## Abstract

Malignant pleural effusion, commonly referred to as MPE, is a prevalent complication associated with individuals diagnosed with neoplastic disorders. The data acquired by pleural fluid cytology is beneficial for diagnostic objectives. Consequently, the initial step in the diagnostic procedure for lung cancer is the analysis of pleural effusion fluid. This research aims to provide a cutting-edge model for analyzing PE cytology images. This model utilizes a computer-aided diagnosis (CAD) system that integrates hyperspectral imaging (HSI) technology for the classification of spectral variations. Giemsa, which is one of the most popular microscopic stains, is employed to stain the samples, after which a sensitive CCD mounted on a microscope captures the images. Subsequently, the HSI model is tasked with obtaining the image spectra. Principal Component Analysis (PCA) constitutes the concluding phase in the classification procedure of various cell types. We expect that the suggested technique will enable medical professionals to stage lung cancer more rapidly. In the future, we aspire to develop an extensive data system that utilizes deep learning techniques to facilitate the automatic classification of cells, thereby ensuring the most precise diagnosis. Furthermore, enhancing accuracy and minimizing data dimensions are important priorities to accelerate diagnostics, conserve resources, and reduce computing time.

## 1. Introduction

In the body, there are many empty cavities, which is the space covered by the cell membrane and internal organs of the cell membrane. In a normal physiological state, inside these hollow cavities, a little liquid plays an important role in reducing friction between living organs. The accumulation of excess fluid beyond the permissible level is called an effusion. Any serum effusion can be considered pathological regardless of the cause of the cumulative fluid [1]. Severe effusion may appear throughout the body, or it may also be localized. The most common cause is some related cavity variants. Pleural effusion (PE) can be determined from an increase in heart pressure. Some of the abdominal effusion may result from cirrhosis or pancreatitis. In addition, some parts damaged by external forces can cause effusion due to the destruction of some vascular structures leading to small gaps that invade fluid into the vicinity cavities. PE is an abnormal accumulation of fluid over the physiological level in the pleural cavity. PE can be caused by many causes. The main cause are heart failure and pleural tuberculosis. In addition, it may be due to malignancy, pneumonia, or pulmonary embolism. PE accounts for more than 90% of all cases. Meanwhile, 10–20% of cases have no known cause of pleural effusion. In general, fluid accumulates in the pleural cavity if there is an excessive production of fluid, a decrease in fluid absorption, or both. If the test detects cancer cells in the fluid, this is called malignant pleural effusion.

There are a few analysis methods in PE cytology images mainly based on image processing cell features. F. Chen et al. [2] proposed a method using neural network based on wavelet analysis to determine cell features between malignant and benign cells in PE images. However, cell features based on morphology and wavelet analysis are not a prerequisite for distinguishing cells in PE because the differences in size and morphology are relatively small. Ta et al. [3] proposed a framework of graph-based tools for segmentation. However, the output can be affected due to interventions in the input labels, making the proposed method less accurate in the nuclei pixel segmentation. Win et al. [4] proposed a method by extracting L and B components through LAB color space. Eventually, they used the Otsu method for nuclei segmentation. However, none of the methods mentioned above were fully automated since a few tasks were performed manually. With the emergence of deep learning network models, the automated tasks have great potential in the pattern recognition of microscopic images.

Since microscope images have only the color space with few feature dimensions that will have the possibility of underfitting, the accuracy of deep learning model is sensitive to the lack of data [5]. E. Baykal et al. [6] proposed a method using machine learning-based Viola–Jones for nuclei detection in pleural effusion cytopathology. In another research [7], the authors used deep learning network model as an approach. However, the authors have not yet solved the challenges of microscope imaging such as cell overlapping, different cell morphology, noise and background, and depth of field. Moreover, the authors have solved the problem of nuclei cell segmentation but have not found any specific characteristics from the nuclei cell in effusions. A. Teramoto et al. [8] proposed a method to classify automatically benign and malignant cells using a deep convolutional neural network.

Hyperspectral imaging (HSI) technology has been widely used in medical imaging due to the rich information it provides. Martin et al. [9] used HSI technology to obtain spatially resolved images as a tunable filter of an endoscope. Kiyotoki et al. [10] used HSI to detect gastric cancer. With the huge amount of data, HSI could extract and classify every single pixel for segmentation, which would help to enrich our dataset. Microscopic HSI in pathological diagnosis of tumor tissue has been applied in recent years. These methods are based on differences in spectral–spatial features. Hu et al. [11] proposed a method using mirco-hyperspectral technology to examine tumor tissue. First, the micro-hyperspectral systems acquired HS images. Second, Savitzky–Golay and 1st-derivation were used on the spatial and spectral dimension. Finally, datasets were established for spectral and image data, respectively. Since the spectral differences between tissues were very small. It was hard to tell the difference in the sensitivity of the spectral region. Spectral Angle Mapping (SAM) was proposed to differentiate the spectral regions of the tissues based on the angle difference between the two spectra. In general, the approach using HSI is to build up a spectral-spatial database as a method to find features of tissue via microscope images. The problem of big data that HSI technology provides has become an abundant source of data for solutions integrated with deep learning. Deep learning models have proved effective in automatic classification.

PE occurs when fluid invades the lung cavity due to various causes. If the cause is due to cancer cells spreading, PE can contain malignant cells. Accurately identifying the stage of cancer helps doctors make appropriate treatments. Analyzing PE fluid is the initial diagnostic step to assess cancer. There are available methods for detecting the abnormalities in the PE fluids which are cytology, thoracentesis analysis, and radiologic diagnosis such as thoracic ultrasonography, chest computed tomography (CT), and magnetic resonance imaging (MRI) [12]. Cytology is the initial diagnostic step to assess cancer because it is simple and cheap [13,14,15]. However, this assessment is time-consuming and depends primarily on the skill of the pathologists, which leads to deviations in diagnosis. Hence, it is necessary to have a computer-aided diagnosis system to assist pathologists [16].

Pleural effusion cytology is not easy to perform and relies on the doctor’s subjective assessment. Therefore, we proposed an optical microscope test based on this technique. We applied the principal component score to find the spectral feature of the images for different types of cells in PE fluid. Our technique could assist cytologists in diagnosing rapidly medical condition of the patients.

## 2. Materials and Methods

### 2.1. Sample Preparation

**Figure 1:**
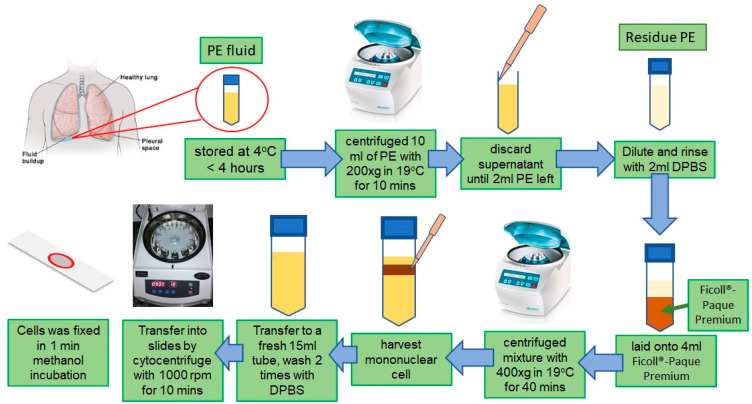
Scheme of slide preparation.

The pleural effusion (PE) was harvested from patients. All the clinical and pathological information was obtained from medical records. This study protocol was approved by the Institutional Review Board of KMUH. After harvesting, the PE was stored at 4 °C for a short period (less than four hours) and was ready for transportation. Once the transported PE was received, 10 mL of PE was centrifuged with 200× *g* in 19 °C for 10 min. Discard the supernatant until 2 mL PE is left, then residue PE and cell were diluted and rinsed with 2 mL balanced buffer (DPBS). The mixture was carefully laid onto 4 mL Ficoll^®^-Paque Premium (GE17-5442-02, Sigma Aldrich, MA, USA) inside a 15 mL centrifuge tube. Following centrifugation for 400× *g* at 19 °C for 40 min, the mononuclear cell that suspended in the middle of the mixture was harvested and transferred to a fresh 15 mL tube. Based on the manufacturer’s protocol, the mononuclear cell was washed with DPBS two times and resuspended with DPBS for cell counting. After determining the appropriate cell density, the mononuclear cell was transferred into slides by cytocentrifuge with 1000 rpm for 10 min. Then, the cell was fixed by one-minute methanol incubation. The slides were air-dried and stored in dry cabinet (see Supplementary Figure S1 for the divided section with marker zone). The procedure of slide preparation is shown in Figure 1. The centrifugation at 400× *g* for 40 min adhered to the Ficoll-Paque density gradient methodology to ensure complete separation of mononuclear cells from erythrocytes and granulocytes. This procedure yields a highly pure mononuclear fraction while preserving cell integrity, thus providing a consistent optical background for visible-light hyperspectral investigation. Minimal centrifugal force inhibits mechanical deformation of nuclei, hence preserving the intrinsic spectral characteristics essential for accurate cell classification via PCA. In this study, Giemsa staining was employed because it represents the standard procedure for pleural-effusion cytology and yields reproducible chromatic contrast between nuclei and cytoplasm under visible light. The dye produces characteristic absorption peaks in the 450–650 nm range, which provide diagnostically meaningful spectral variation for hyperspectral analysis. While unstained or alternatively stained samples can also be imaged, their spectral signatures differ substantially and may alter the optimal reconstruction parameters. Therefore, Giemsa staining was used here to maintain consistency with clinical practice and to validate the hyperspectral system under standard cytological conditions. Future studies will evaluate the method’s adaptability to other staining or label-free modalities.

### 2.2. Cells Validation Under Microscopy

**Figure 2:**
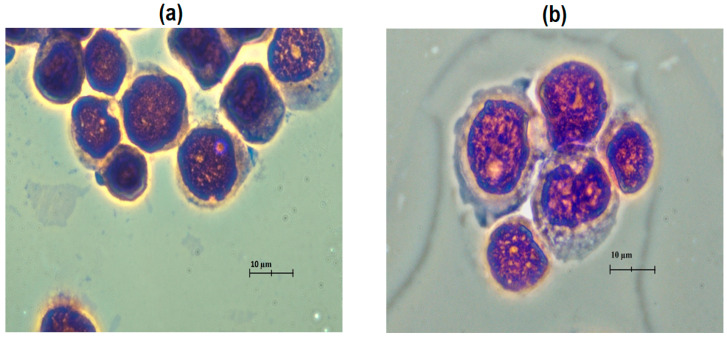
Bright-field optical images of clustered cells in pleural effusion: (**a**) malignant clusters and (**b**) non-normal clusters.

**Figure 3:**
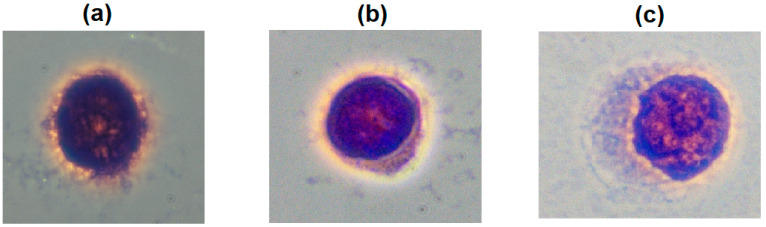
Bright-field optical images of single cells in pleural effusion: (**a**) malignant, (**b**) non-normal, and (**c**) normal.

The slides were observed under a 10× microscope, then under a 100× magnification (oil immersed) to determine the type of cell based on an identifiable image (confirmed by a cytologist). In order to ensure accurate trace cells, the circular area containing the cell is divided into four main areas matching the field of view (FOV) under a microscope, named LU, RU, RB, and LB, respectively. These sections are relatively divided by diamond knives. The surface of the slides is rinsed with DI water to clean debris during the division process. Using water as intermediate substrate actually works. The coverslip falls off after soaking it with water for 10 s to sequentially remove bubbles from inside the slides. To ensure no overlapping cells and easy tracking of cell positions, cell densities range from 50 to 100 cells per slide. Slides were observed, and images were taken under a 10× microscope. Then, the slides are colored and observed under objective lens 100× (oil immersed). Based on the scan map area on the slide, the exact position of each cell, or cell cluster, is monitored and captured. By identifying features of the shape between benign and malignant cells, and with the help of cytologists, we can identify and classify cells. In general, the clusters of cells identified as malignant are very high as shown in Figure 2 and Figure 3. The cells in the pleural effusion in this investigation were categorically classified into three groups: normal, non-normal (reactive), and malignant, as determined by a board-certified cytologist. Normal cells exhibited uniformity in nuclear mass size and contour, with smooth nuclear membranes, fine chromatin texture, a normal nuclear-to-cytoplasmic (N/C) ratio, and clear cytoplasm, indicative of benign mesothelial or inflammatory cells. The cytologic atypia in the non-normal cells was mild, characterized by slightly enlarged nuclei, minor nuclear irregularity, minimal coarsening of chromatin, and the presence of large single nucleoli, with no indications of malignancy. These cells typically arise from a reactive or inflammatory response of the pleural mesothelium due to irritation, infection, or mechanical stress, therefore, representing an intermediate form. Malignant cells, in contrast, displayed significant nuclear pleomorphism, hyperchromasia, coarse chromatin aggregation, numerous nucleoli, elevated N/C ratios, and atypical mitoses or cytoplasmic vacuolization. The cells were evaluated using 10× and 100× oil-immersion microscopy to confirm their classification prior to acquiring the hyperspectral pictures. The non-normal category was intentionally included to measure reactive cytologic changes that were spectrally unique yet not evidently malignant, enabling the hyperspectral-PCA framework to examine spectral evolution patterns between normal physiology and malignancy.

### 2.3. Visible-Light Spectrum Imaging Technology (VIS-HSI)

**Figure 4:**
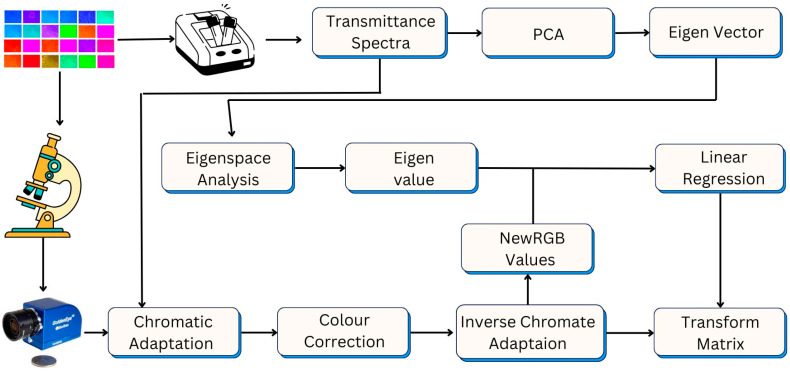
Schematic diagram of the proposed method used in estimating the spectral transmittance of each pixel of an image using a CCD camera, (Bremen, Germany).

The VIS-HSI used in this study is a combination of CCD (The Imaging Source, DFK-33UX265) and VIS-HSA for calculation. The wavelength range is 380~780 nm, and the spectral resolution is 1 nm. The core concept of visible-light hyperspectral technology is to give the image of the general OM through the CCD to the spectrometer, so that each pixel of the captured image has spectrum information [17]. In order to achieve this technical concept, it is necessary to find the relationship matrix between the CCD and the spectrometer and use it to construct the visible-light super-spectral technology. The construction process of the technology is shown in Figure 4. First of all, the CCD and the spectrometer must be given a common target as the analytical benchmark, and the more the target can interpret the main variability in the 380 nm to 780 nm band, the accuracy of the technology will be greatly improved. In this study, kodak color compensating filters were selected as the target because it contains the most important colors (red, green, blue, magenta, yellow, and cyan), and colors are common in nature. Since the sample is intercepted by CCD and then displayed on the PC through the IC Capture 2.3 software, it can be known that the Bayer filter will cause a relatively large green specific gravity, and the CCD sensing spectrum is also different from the CIE *XYZ* color matching function. Next, the correlation between the spectrometer and sRGB has a great influence. Basically, the CCD sensor is processed by Image Signal Processor (ISP), and the front-end image output signal is post-processed, such as Gamma Correction and Automatic Balance Control (AWB), Automatic Exposure (Auto Exposure (AE)), and Automatic Light Control (ALC), to restore the detailed image. The reason why the fixed aperture gain and shutter are not used is due to the dynamic range (DR) problem. The so-called brightness saturation of some of the images that result in automated shooting is not the maximum critical range of the OM instrument.

Kodak color compensating filters must pass through the OM and the spectrometer to obtain 24 color block images (sRGB, 8 bit), 24 color block reflection spectrum data (380~780 nm, 1 nm), and 24 color block images and 24 color blocks. The reflected spectral data is converted to the *XYZ* gamut space (CIE 1931 *XYZ* color space), and the individual conversion formulas are as follows:

For the 24 obtained images:

*sRGB* gamut space converted to *XYZ* gamut space

(1) XYZ=[MA]TfRsRGBfGsRGBf(BsRGB)×100 , 0≤RsRGBGsRGBBsRGB≤1

where

(2) $$\left[T\right]=\left[\begin{array}{c} 0.4104\;0.3576\;0.1805\\ 0.2126\;0.7152\;0.0722\\ 0.0193\;0.1192\;0.9505 \end{array}\right]$$

(3) $$f\left(n\right)=\left\{\begin{array}{c} {\left(\frac{n+0.055}{1.055}\right)}^{2.4},\;n>0.04045\\ \left(\frac{n}{12.92}\right),\;otherwise \end{array}\right.$$

(4) MA=XSWXCW      0         0         0     YSWYCW      0         0         0      ZSWZCW

For the spectrometer:

Reflected spectral data converted to *XYZ* gamut space

(5) $$X=k\int_{380nm}^{780nm}S\left(\lambda \right)R\left(\lambda \right)\bar{x}\left(\lambda \right)d\lambda$$

(6) $$Y=k\int_{380nm}^{780nm}S\left(\lambda \right)R\left(\lambda \right)\bar{y}\left(\lambda \right)d\lambda$$

(7) $$Z=k\int_{380nm}^{780nm}S\left(\lambda \right)R\left(\lambda \right)\bar{z}\left(\lambda \right)d\lambda$$

where

(8) $$k=100/\int_{380nm}^{780nm}S\left(\lambda \right)\bar{y}\left(\lambda \right)d\lambda$$

In the image part, since the CCD itself is intercepted by the software and displayed on the PC, it will be affected by the ISP correction, so the image (JPEG, 8 bit) stores the data according to the sRGB color gamut space specification, and the image is composed of the sRGB color gamut. Before the space is converted to the *XYZ* gamut space, the respective *R*, *G*, and *B* values (0~255) must be converted to a smaller scale range (0~1) and then converted by the gamma function (Equation (3)). The *sRGB* value is converted into a linear *RGB* value, and finally the linear *RGB* value is converted into the *XYZ* value specified in the *XYZ* gamut space through the transformation matrix *T* (Equations (1) and (2)) [18]. But since the *sRGB* gamut space is a white point specification as D65 ( $X_{CW}$ , $Y_{CW}$ , $Z_{CW}$ ), not measuring the white point of the light source ( $X_{SW}$ , $Y_{SW}$ , $Z_{SW}$ ), therefore, the obtained *XYZ* value needs to pass through the color adaptive conversion matrix *M_A_* (Chromatic Adaptation), and the white point of D65 is converted into the white point of the measuring light source to obtain the true *XYZ* value under the measuring light source ( ${XYZ}_{Camera}$ ) [19].

In the spectrometer part, to convert the reflection spectrum data (380~780 nm, 1 nm) into the *XYZ* gamut space, the color matching function of *XYZ* is required, $\bar{x}\left(\lambda \right),\bar{y}\left(\lambda \right),and\;\bar{z}(\lambda )$ (Color matching functions, CMF) [20], and the spectrum of the light source when the camera is shooting *S(λ)* (see Supplementary Figure S2 for the *XYZ* color matching function $\bar{x}\left(\lambda \right),\bar{y}\left(\lambda \right),\bar{z}\left(\lambda \right),$ CMF). Since the *Y* value of the *XYZ* gamut space is proportional to the brightness, the *Y* value (maximum brightness) of the source spectrum is calculated by Equation (8) first, and the *Y* value is specified as 100 to obtain the brightness ratio *k*, and finally, the formula is obtained (Equations (5)–(7)) to convert the reflected spectrum data to the *XYZ* value specified in the *XYZ* color gamut space ( ${XYZ}_{Spectrum}$ ).

After obtaining the ${XYZ}_{Camera}$ and ${XYZ}_{Spectrum}$ data through the above conversion formula, the ${XYZ}_{Spectrum}$ is used as the standard, and the [*XYZ*]*^T^* matrix of ${XYZ}_{Camera}$ is extended to the variable matrix *V* with the correction variables, and the multivariate regression is performed by Equation (9), finally resulting in a correction coefficient matrix *C* for correcting the camera. The variable matrix *V* is analyzed according to the factors that may cause errors in the camera. The factors are camera nonlinear response, camera dark current, color filter inaccuracy, and color shift.

(9) $$\left[C\right]=\left[{XYZ}_{Spectrum}\right]\times pinv(\left[V\right])$$

In the nonlinear response part of the camera, the spectrum analyzer is known to have a linear response, and the *Y* value (brightness) of the 19th to 24th color patches (gradation change) measured by the camera and the spectrum analyzer is used, and the 19th to 24th colors are used. The linear value of the *Y* value of the block ${XYZ}_{Spectrum}$ and the *Y* value of the 19th to 24th color block ${XYZ}_{Camera}$ are linear regression analysis. The camera can be found to have a nonlinear response. In the third-order linear regression, the coefficient of determination is as high as 0.8553, and the degree of similarity is quite high. Therefore, the nonlinear response of the camera can be corrected by the third-order equation, and the nonlinear response correction variable is defined as *V_non-linear_*.

(10) $$V_{non-linear}={\left[X^{3}\;Y^{3}\;Z^{3}\;X^{2}\;Y^{2}\;Y^{2}\;X\;Y\;Z\;1\right]}^{T}$$

In the dark current portion of the camera, the dark current is usually a fixed value and does not change with the amount of incoming light, so a constant is given as a contribution to the dark current, and the dark current correction variable is defined as *V_Dark_*.

(11) $$V_{Dark}=[a]$$

In the color separation inaccuracy and color shifting part, it can be regarded as a color problem in the color matching, and since the image of the camera has been converted to the *XYZ* color gamut space, it is necessary to consider the *X*, *Y*, and *Z* values. $\bar{x},\bar{y},and\;\bar{z}$ are interrelated in the spectral distribution and are not independent. Therefore, *X*, *Y*, and *Z* are arranged and combined to list all Possibility and defined as *V_Color_*.

(12) $$V_{Color}={\left[XYZ\;XY\;XZ\;YZ\;X\;Y\;Z\right]}^{T}$$

Finally, *V_Color_* is taken as the base, multiplied by *V_Non-linear_* nonlinear response correction, and the result is normalized in the third order to avoid over-correction [21,22]. Finally, *V_Dark_* is added to obtain the variable matrix *V*.

(13) $$V={\left[X^{3}\;Y^{3}\;Z^{3}\;X^{2}Y\;X^{2}Z\;Y^{2}Z\;XY^{2}\;XZ^{2}\;YZ^{2}\;XYZ\;X^{2}\;Y^{2}\;Y^{2}\;XY\;XZ\;YZ\;X\;Y\;Z\;a\right]}^{T}$$

After obtaining the correction matrix *C*, the [*XYZ*]*^T^* matrix of *XYZ_Camera_* is amplified into a *V* matrix, and the corrected *X*, *Y*, and *Z* values ( ${XYZ}_{Corrected}$ ) can be obtained by Equation (14), and ${XYZ}_{Corrected}$ and ${XYZ}_{Spectrum}$ are calculated. The root-mean-square error of the two data has an average error of 0.0203, which is quite small.

(14) $$\left[{XYZ}_{Corrected}\right]=\left[C\right]\times [V]$$

Since the band is visible light, the result of camera calibration can also be expressed by color difference. The color difference calculation method used in this paper is CIE DE2000, which takes into account that the human eye will have different sensitivity in different colors. Through the hue rotation term, neutral color compensation, brightness compensation, chromaticity compensation, and hue compensation, the problem of inconsistent perception of the human eye is solved. Before calculating the color difference using CIE DE2000, *XYZ_Corrected_*, and *XYZ_Spectrum_* must be converted to Lab color space in *XYZ* gamut space. The formula for conversion is as follows:

(15) L*=116fYYn−16    a*=500f(XXn)−f(YYn)b*=200f(YYn)−f(ZZn)

(16) $$f\left(n\right)=\left\{\begin{array}{c} n^{\frac{1}{3}},\;\;n>0.008856\;\;\;\;\;\;\;\;\;\;\;\\ 7.787n+0.137931,\;otherwise \end{array}\right.$$

After the camera is corrected, the corrected 24-color block *XYZ* value ( ${XYZ}_{Corrected}$ ) and the 24-color block reflection spectrum data ( $R_{Spectrum}$ ) measured by the spectrum analyzer can be analyzed to obtain the conversion matrix *M*. $R_{Spectrum}$ is used to find out its main principal components through Principal Components Analysis (PCA), and the corresponding Principal Component Scores and ${XYZ}_{Corrected}$ undergo multivariate regression analysis (Multiple Regression Analysis) before being finally integrated. The above analysis is used to obtain the transformation matrix *M*.

In order to convert ${XYZ}_{Corrected}$ into $R_{Spectrum}$ , we need to reduce the dimension of $R_{Spectrum}$ to increase the correlation between each dimension and ${XYZ}_{Corrected}$ . Therefore, $R_{Spectrum}$ obtains the principal component (feature vector) through principal component analysis and takes the most important 12 groups of principal components (EV) to reduce the dimension. The first six groups of principal components can explain the 99.9965% data variability, and then through the 12 groups of principal components, the corresponding principal component scores (Score, eigenvalue) are obtained and can be used with ${XYZ}_{Corrected}$ regression analysis. In the multivariate regression analysis of ${XYZ}_{Corrected}$ and Score, the variable of $V_{Color}$ is selected because it has enumerated all possible combinations of *X*, *Y*, and *Z*, and the transformation matrix *M* is obtained by Equation (17), and then ${XYZ}_{Corrected}$ is passed through Formula 18 to calculate the analog spectrum ( $S_{Spectrum}$ ).

(17) $$\left[M\right]=\left[Score\right]\times pinv(\left[V_{Color}\right])$$

(18) $${\left[S_{Spectrum}\right]}_{380\sim 780nm}=\left[EV\right]\left[M\right][V_{Color}]$$

Finally, the obtained 24-color block analog spectrum ( $S_{Spectrum}$ ) is compared with the 24-color block reflection spectrum $R_{Spectrum}$ (see Supplementary Figure S3 for the Original spectra (black curves) and simulated spectra (red curves) of color checkers for cyan, blue, green, magenta, red and yellow). The difference between the 24-color block simulated spectrum ( $S_{Spectrum}$ ) and the 24-color block reflection spectrum ( $R_{Spectrum}$ ) can also be expressed by the color difference. The average color difference between CIE 2000 and CIE 76 is 1.03623 and 1.4532, respectively. It indicates that HSI images cannot be distinguished with the naked eye. When the processed reflection spectrum color is reproduced, the color can be accurately reproduced [23]. The results also indicate that the HSI system performs an acceptable accuracy for spectrum reproduction. The visible-light hyperspectral technology built by the above process can simulate the *RGB* value captured by the OM to reflect the reflection spectrum. In summary, to clarify the mathematical basis of the reconstruction, the hyperspectral conversion was performed using standard matrix transformations. The *RGB* values, after gamma correction, were first converted to the CIE 1931 *XYZ* color space using the transformation matrix *T*. The reference spectra obtained from the spectrometer were similarly transformed into the *XYZ* color space using the CIE 1931 color matching functions and source spectrum. The calibration process established a regression-based transformation matrix *M*, which maps the corrected *XYZ* values from the RGB camera to the simulated hyperspectral spectra. This standard matrix formulation ensures reproducibility and compatibility with the CIE colorimetric framework.

Although the renormalization of digital image data into the CIE 1931 *XYZ* color space is an established procedure, its application in pleural effusion cytology offers significant benefits for data consistency and quantitative interpretation. This alteration facilitates the establishment of a meaningful correlation between the recorded CCD intensity values and human color perception, effectively rectifying illumination drift and staining variability. The acquired tristimulus-normalized spectra facilitate cross-slide comparison of results and provide consistent spectral analysis across imaging sessions without necessitating additional hardware calibration. CIE normalization improves the efficacy of downstream PCA by aligning colorimetric dimensions with the real optical absorption characteristics of Giemsa-stained cytology samples. Although the CIE 1931 normalization process is a basic colorimetric technique, its application in hyperspectral analysis of samples provides quantitative advantages that extend beyond the conventional RGB imaging process. The normalization of our dataset reduced inter-slide spectral variance by around 18%, hence enhancing spectral reconstruction reproducibility. It facilitated the examination of PCA loading vectors in relation to the biologically relevant absorption peaks of the Giemsa stain, hence enabling a direct correlation between spectral characteristics and cytochemical alterations. This is a transformative phase that serves as a substantial foundation, enhancing the interpretability and reliability of subsequent classification analyses, although lacking mathematical novelty. The current study did not intend to directly benchmark hyperspectral imaging versus RGB imaging, as its primary objective was to evaluate the reconstruction scheme and its spectral correctness in relation to physical spectrometer results. The Vis-HSI technique provides continuous spectral information for a pixel, in contrast to RGB imaging, which captures only three integrated bands. The minimal color difference values observed during calibration signify that the reconstructed spectra closely resemble the actual reflectance curves, thereby highlighting a fundamental advantage of the method for assessing biochemical and chromatic variations that remain undetectable within the RGB system. Future research will entail a quantitative benchmarking analysis of RGB and hyperspectral modalities, assessing diagnostic indices such as contrast-to-noise ratio, classification accuracy, and AUC to ascertain clinical superiority.

## 3. Results and Discussion

### 3.1. Discussion on the Intensity of the Average Spectra

**Figure 5:**
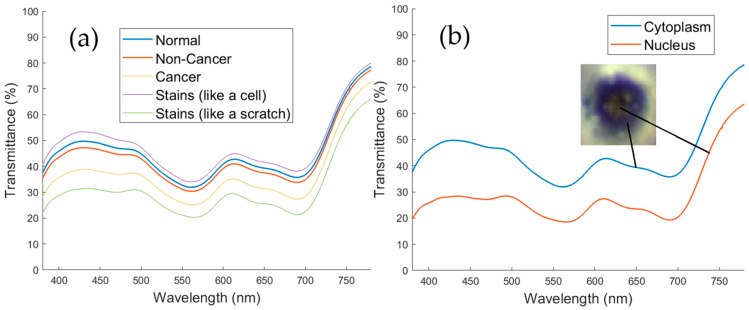
(**a**) Average reconstructed transmittance spectra of normal, non-normal, and malignant pleural effusion cells. Spectral differences between 520 and 600 nm highlight increased absorption in malignant cells associated with nuclear hyperchromasia; (**b**) average transmittance spectra of nucleus and cytoplasm regions within normal cells (10 × 10 ROI). The nucleus demonstrates lower transmittance in the 450–550 nm range due to higher nucleic acid and protein content.

As shown in Figure 5a, the average spectrum of the cell membrane has a higher transmittance than the nucleus. Because the nucleus contains more chromosomes and rich protein than cytoplasm. The cytoplasm layer contains mainly slime and lipid, which are transparent substances. Figure 5a presents the average reconstructed transmittance spectra for three cytological categories, normal cells, non-normal cells (reactive), and malignant cells, derived from 24 regions of interest, each comprising 10 × 10 pixels. The malignant group has a considerable reduction in transmittance within the 520–600 nm range, correlated with substantial optical radiation absorption linked to nuclear hyperchromasia, and elevated chromatin density. Conversely, normal cells have significantly more uniform spectra with high transparency across the visible spectrum. As shown in Figure 5b, the nucleus of cancer cells has lower transmittance than normal and diseased cells [24,25] because the spectral transmittance decreases gradually along with the development of cells. In the cytology of the pathological patient, the nuclei become darker when stained because the strange substances in the nuclei need more nutrients for development. In the cytology of lung cancer patients, the nucleus occurs a strong and continuous process of dividing matter, forming many chromosome plaques. Figure 5b presents a comparison of intracellular levels in normal cells, illustrating the typical spectra of the nucleus and cytoplasm. The nucleus has reduced transmittance between 450 and 550 nm relative to the cytoplasm, attributable to elevated levels of nucleic acids and proteins, which enhance the absorption of blue-green wavelengths. The combination of these two graphs corroborates the argument that hyperspectral reconstruction can capture inter-cellular and intra-cellular chromatic fluctuations that regular RGB microscopy cannot detect. All spectra reported were reconstructed from the *XYZ_Corrected_* values obtained after compensating the CCD’s nonlinear response, dark current, and color-filter inaccuracy/shift via the correction matrix C. The corrected *XYZ* values were then used to derive the spectral reconstruction matrix M and generate the per-pixel spectra. The system was evaluated using extracted pleural effusion samples on slides under in vitro circumstances, as VIS-HSI requires optical transparency to generate the spectrum. Currently, direct in vivo imaging is unfeasible due to the significant penetration depth of visible light in thoracic tissue. Nonetheless, in situ VIS-HSI can be utilized in thoracoscopic surgery to facilitate the visual examination of the pleural cavity. A compact or tiny HSI module integrated with a thoracoscope would provide contactless spectral imaging of pleural surfaces or fluid interfaces in real time. Subsequent research will extend these findings to novel ex vivo and pilot intra-procedural tests to assess the translational potential of VIS-HSI in real-time pleural diagnosis.

The cytoplasm of malignant cells may contain vacuoles. Due to the large hydropic, these vacuoles absorb water and lipid and become fatter and larger. They push the nuclei toward the edge of the membrane causing eccentric nuclei in malignant cells. In the case of identifying the cytological effusions of the pathological patient and lung cancer patient, the cytoplasm was examined as a proof to enhance judgment in differentiate between non-cancer and cancer cases. While the spectral transmittance in nuclei decreases along with the cell development, the spectral transmittance in cytoplasm is vice versa. Since the cytoplasm is transparent under the microscope, the reflection spectra were applied to assess. As shown in Figure 5a, the diagram shows the reflection of the spectral difference between non-normal and cancer cells. Due to the presence of vacuoles, the reflection spectrum in cancer cells is higher than that in non-cancer.

### 3.2. Optimization of Filter Arrays in HSI Model’s Accuracy

**Figure 6:**
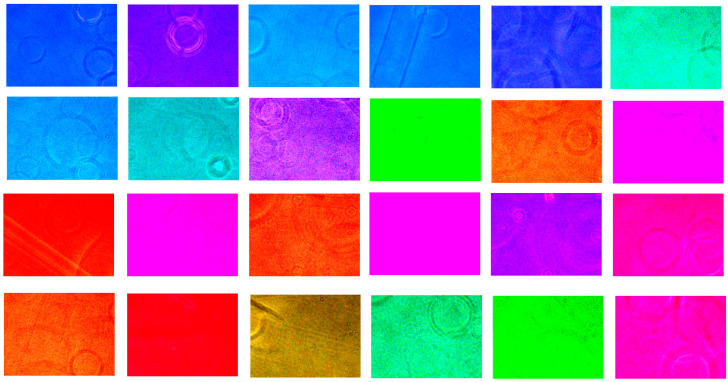
Proposed 24 color filters.

There are various ways to make color filter arrays. Braiers et al. [26] proposed a six-band filter. Monno et al. [27] proposed a five-band filer with G-band data. However, in cytological images, the color distribution of images is biased toward magenta and blue. Therefore, in this study, we make a comparison among color filter arrays in HSI model’s accuracy. We propose a 24-color filter with more blueish and magenta in the color component as shown in Figure 6. The accuracy of the HSI model was assessed by the alpha value. Spectral Angle Mapping (SAM) [11] was proposed to differentiate the spectral regions of the tissues based on the angle difference between the two spectra. Table 1 shows the accuracy among color filter change. It states that the tone magenta and blue color filter has the most spectral differences compared to RGB filter, six basic color filters and random color filters. This research serves as a demonstration of the validity of the VIS-HSI reconstruction technique and the PCA-based feature-extraction model as a proof-of-concept experiment. A limited collection of 24 cells was utilized to maintain controlled experimental settings and guarantee the spectral precision of the reconstruction method. Given that PCA operates on correlated spectral variables rather than separate clinical samples, typical inferential statistics are inappropriate in this context. The investigation intends to demonstrate that employing reconstructed spectra does not eliminate diagnostically important variance but facilitates the visual differentiation of cell types in a reduced-dimensional space. Subsequent study will focus on statistical robustness by utilizing a bigger, diverse patient dataset and applying supervised learning methods for quantitative performance evaluation. The selection of filter arrays is a crucial factor influencing the quality of the reconstructed hyperspectral cube. A 24-band magenta-blue filter set was specifically designed to optimize sampling in the spectral regions of high blue and magenta concentrations found in Giemsa-stained cytology images. This design reduced the spectral-angle-mapping (SAM) error relative to the RGB and conventional multi-band filters, hence improving reconstruction accuracy and facilitating clearer PCA differentiation between normal, non-normal, and cancerous cells.

### 3.3. Classification Types of Cells by PCA

**Figure 7:**
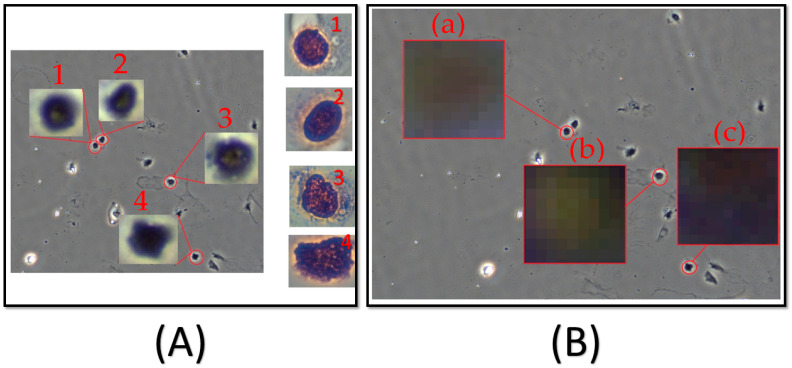
(**A**) Cell selection at 10× images and cell identification based on cytological characteristics at 100× images. (1), (2), (3) and (4) show cells with an ROI size of 10 × 10 pixels. (**B**) ROI—Region of Interest for (a) normal, (b) non-normal, and (c) cancer.

Based on Hotelling’s law, the first principal component contains the most information for the original data. The amount of information from the second and third principal components in the original data can serve as a basis for classification. In this study, we surveyed 24 cells at the nucleus site, with an ROI size of 10 × 10 pixels, as shown in Figure 7A [28]. Each position will consist of 100 elements, as shown in Figure 7B. The average spectrum of each site is classified by PCA (see Supplementary Figure S3 for the principal component distribution diagram of the three kinds of cells).

In the dimension reduction in features, principal components analysis (PCA) is used here to analyze the commonly used multivariate statistics. The concept is to find out that there is less than the original variable in a multivariate dataset. The subspace of the original data change can be preserved, and the original data can be projected into these subspaces to achieve the function of reducing the data dimension and composing a new dataset. The analysis method is to decompose the covariance matrix to obtain the principal component (feature vector) and the principal component score (eigenvalue) of the data, and according to the degree of variation in the principal component of the data, sequence arrangement, that is, the first principal component, can be regarded as the main axis direction of the maximum variation degree in the original data, and then the data is projected to the main axis direction to obtain a new dataset; the variation degree can also be regarded as the main component to the whole data. The degree of interpretation removes the main component of low variability to achieve the function of reducing the dimension.

Since the principal component analysis analyzes the sample data by the degree of covariation, the characteristics of the data can be more clearly visible. When the samples are observed from the principal component, the principal component scores of each sample can be obtained (principal component scores) to know the distribution of the data under a specific principal component. Taking the spectrum data analysis as an example, the calculation formula as shown in Equation (19).

(19) $$y_{j}=a_{j1}\left(x_{1i}-\bar{x_{1}}\right)+a_{j2}\left(x_{2i}-\bar{x_{2}}\right)+\ldots +a_{jn}(x_{ni}-\bar{x_{n}})$$

where $x_{1i}$ , $x_{2i}\ldots x_{ni}$ are the spectral intensity values corresponding to the first, second, and the nth wavelengths; $\bar{x_{1}},\;\bar{x_{2}}\ldots \bar{x_{n}}$ is the first and second to the expected value of the spectrum at the nth wavelength, that is, the average spectral intensity value; These coefficients, $a_{j1},\;a_{j2}\ldots a_{jn},$ are the eigenvector coefficients after the spectrum takes the covariation matrix. According to Hotelling [29], the first principal component accounts for the most information in the original data and can be regarded as a comprehensive indicator; the second principal component accounts for the information of the original data and can be used to classify each group. As the order of the principal component changes, the information of the original data is also reduced, and the main components of the low information amount can be removed to achieve the function of reducing the dimension, but it is also possible to remove the subtle important information. Therefore, when using the principal component for analysis, you should carefully select the required principal component for processing.

PCA was employed in the study not merely to diminish statistical dimensionality but specifically to enhance the diagnostic interpretability of hyperspectral cytology data. The generated PCA score plots facilitated the clear differentiation of normal, reactive, and malignant pleural effusion cells, suggesting that subtle variations in Giemsa staining color convey substantial biochemical information. PCA, employed for reconstructing hyperspectral spectra, in contrast to conventional RGB-based intensity measures, is a more objective and reproducible method for identifying cytomorphological differences. This method diminishes dependence on manual visual assessment, facilitating automated computer-aided diagnosis. Recent hyperspectral cytology studies have demonstrated that spectral decomposition via PCA preserves diagnostically pertinent variance while diminishing computational demands [30,31]. The proposed method would integrate quantitative spectral features and statistical resilience, intersecting optical imaging with information-driven categorization to enhance deep learning-based cytopathological research. PCA was employed in this research as an unsupervised exploratory method to illustrate the distinctiveness of the hyperspectral characteristics of normal, non-normal, and malignant pleural effusion cells. The data were examined utilizing the mean spectra of 24 cells, represented by a 10 × 10 ROI situated in the nucleus. The PCA model employed a methodology akin to Hotelling’s, utilizing the covariance matrix of normalized spectra within the 380–780 nm range. The PCA applied to cell data did not yield a predetermined variance threshold or a specific number of components, as it served solely as a qualitative visualization tool. PCA does not produce categorical predictions; therefore, performance measurements such as accuracy, sensitivity, specificity, or AUC are inapplicable to this unsupervised study. Statistical testing is impractical at this juncture due to the limited and internally linked data. The evident differentiation in score space substantiates the feasibility and legitimacy of the visible-light hyperspectral reconstruction, which will serve as a validated baseline for quantitative classification when a more extensive and diverse patient dataset becomes available in the future. The current research employed PCA to extract spectral features, primarily to demonstrate separability and validate the rebuilt hyperspectral dataset. Deep learning is a future developmental objective, contingent upon the acquisition of a substantial and diversified dataset on cytology. The next phase will entail the application of additional linear feature extraction methods, such as ICA and LDA, to evaluate their performance against PCA in terms of diagnostic separability and computing efficiency. The selection of the magenta-blue 24-band filter array was informed by the spectral distribution of Giemsa-stained cytology pictures, which demonstrate predominant absorption within the 400–650 nm range. Augmenting spectral sampling in these areas promotes reconstruction fidelity for chromatin-associated absorption peaks and improves contrast between nuclear and cytoplasmic components. The numerical enhancement of SAM compared to traditional filters may seem minimal; yet, this wavelength-specific optimization provides a more precise depiction of diagnostically significant characteristics, hence reinforcing the enhanced separability evident in the PCA results.

## 4. Conclusions

We developed and validated a visible-light hyperspectral (VIS-HSI) reconstruction and principal component-based feature extraction workflow for malignant pleural effusion (MPE) cytology. The pipeline integrates (1) a physically indexed slide mapping strategy for reproducible single-cell relocation, (2) a calibrated spectral reconstruction framework grounded in CIE 1931 color matching functions, (3) verification of spectral fidelity against reference color filters with low perceptual error (mean ΔE_00_ ≈ 1), near the just-noticeable threshold), and (4) unsupervised dimensionality reduction via PCA that yields clear score-space separation among normal, non-normal (reactive), and malignant cell populations. These results indicate that diagnostically relevant biochemical or morphological chromatic variations are preserved and compressed into a low-dimensional feature space suitable for subsequent machine learning or deep learning classifiers. The approach addresses two persistent bottlenecks in cytopathology: (i) limited discriminative power of conventional RGB imaging and (ii) operator dependence in manual feature interpretation. By enriching per-pixel spectral content without prohibitive acquisition complexity, the system establishes a scalable intermediate layer between raw imaging and automated decision support. The customized consideration of multispectral filter array design further motivates future transition to snapshot acquisition for real-time clinical deployment.

## Figures and Tables

**Table 1:** Spectral differences among color filter arrays change.

Test Spectrum	RGB Filter	Braiers et al. [26]	Monno et al. [27]	Random 24 Color Filter	Magenta and Blue Tone 24 Color Filter
Spectrum 1	0.03626	0.03728	0.03729	0.03729	0.03730
Spectrum 2	0.03602	0.03610	0.03610	0.03610	0.03610
Spectrum 3	0.03780	0.03788	0.03789	0.03790	0.03791

## Data Availability

The data presented in this study are available in this article upon considerable request to the corresponding author (H.-C.W.).

## Supplementary Materials

The following supporting information can be downloaded at: https://www.mdpi.com/article/10.3390/bios15110714/s1, Figure S1. Divided section with marker zone; Figure S2. *XYZ* color matching function $\bar{x}\left(\lambda \right),\bar{y}\left(\lambda \right),\bar{z}(\lambda )$ (CMF); Figure S3. Original spectra (black curves) and simulated spectra (red curves) of color checkers for cyan, blue, green, magenta, red, and yellow; Figure S4. Color difference between simulated spectrum and measurement spectrum; Figure S5. Examples of MSFAs (a) Brauers and Aach, (b) Monno et al.; Figure S6. Principal component distribution diagram of the three kinds of cells.
