# Supplementary material for: Visible-Light Hyperspectral Reconstruction and PCA-Based Feature Extraction for Malignant Pleural Effusion Cytology
Source: Biosensors (Basel). 2025 Oct 28;15(11):714. doi: 10.3390/bios15110714 (PMC12650489; doi:10.3390/bios15110714)
Supplement: Supplementary file 1 [file biosensors-15-00714-s001.zip › biosensors-3858405-supplementary.pdf]

biosensors

MDPI

Supplementary

Visible-Light Hyperspectral Reconstruction and PCA-Based Feature Extraction for Malignant Pleural Effusion Cytology

Chun-Liang Lai [1,2], Kun-Hua Lee [3,4], Hong-Thai Nguyen [4,5], Arvind Mukundan [4,6,7], Riya Karmakar [4,7], Tsung-Hsien Chen [8], Wen-Shou Lin [9,*] and Hsiang-Chen Wang [4,10,*]

1 Division of Pulmonology and Critical Care, Department of Internal Medicine, Dalin Tzu Chi Hospital, Buddhist Tzu Chi Medical Foundation, No. 2, Minsheng Road, Dalin, Chiayi 62247, Taiwan; laicl.dalin@gmail.com
2 School of Medicine, Tzu Chi University, 701 Zhongyang Rd., Sec. 3, Hualien 97004, Taiwan
3 Department of Trauma, Changhua Christian Hospital, No. 135, Nanxiao St., Changhua City 50006, Taiwan; 88847@cch.org.tw
4 Department of Mechanical Engineering, National Chung Cheng University, 168, University Rd., Min Hsiung, Chiayi 62102, Taiwan; hongthai.nguyen@tnut.edu.vn (H.-T.N.); arvindmukund96@gmail.com (A.M.); karmakarriya345@gmail.com (R.K.)
5 Department of Mechanical Engineering, Thai Nguyen University of Technology, No. 666, Street 3/2, Thai Nguyen City, Vietnam
6 Department of Biomedical Imaging, Chennai Institute of Technology, Sarathy Nagar, Chennai 600069, India
7 School of Engineering and Technology, Sanjivani University, Sanjivani Factory, Singnapur, Kopargaon, Maharashtra 423603, India
8 Department of Internal Medicine, Ditmanson Medical Foundation, Chia-Yi Christian Hospital, Chiayi 60002, Taiwan; cych13794@gmail.com
9 Neurology Division, Department of Internal Medicine, Kaohsiung Armed Forces General Hospital, 2, Zhongzheng 1st.Rd., Lingya District, Kaohsiung City 80284, Taiwan
10 Technology Development, Hitspectra Intelligent Technology Co., Ltd., Kaohsiung 80661, Taiwan
* Correspondence: linvincent1009@gmail.com (W.-S.L.); hcwang@ccu.edu.tw (H.-C.W.)

S1. Sample Preparation

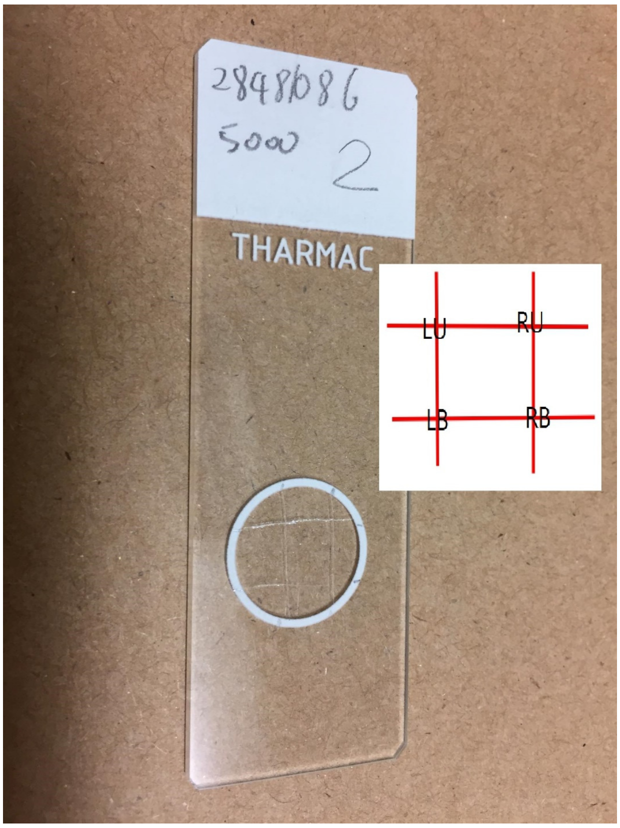

Figure S1. Divided section with marker zone.

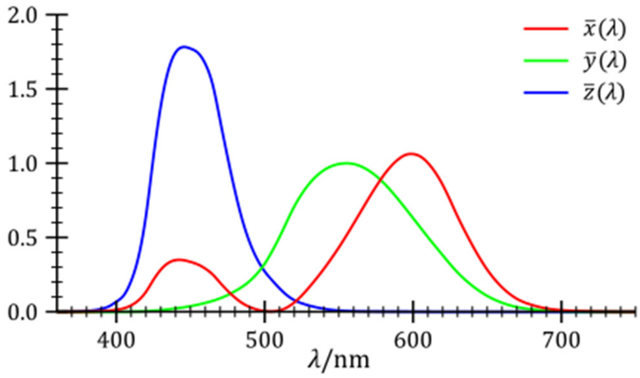

Figure S2. XYZ color matching function$\bar{x}(\lambda), \bar{y}(\lambda), \bar{z}(\lambda)$ (CMF).

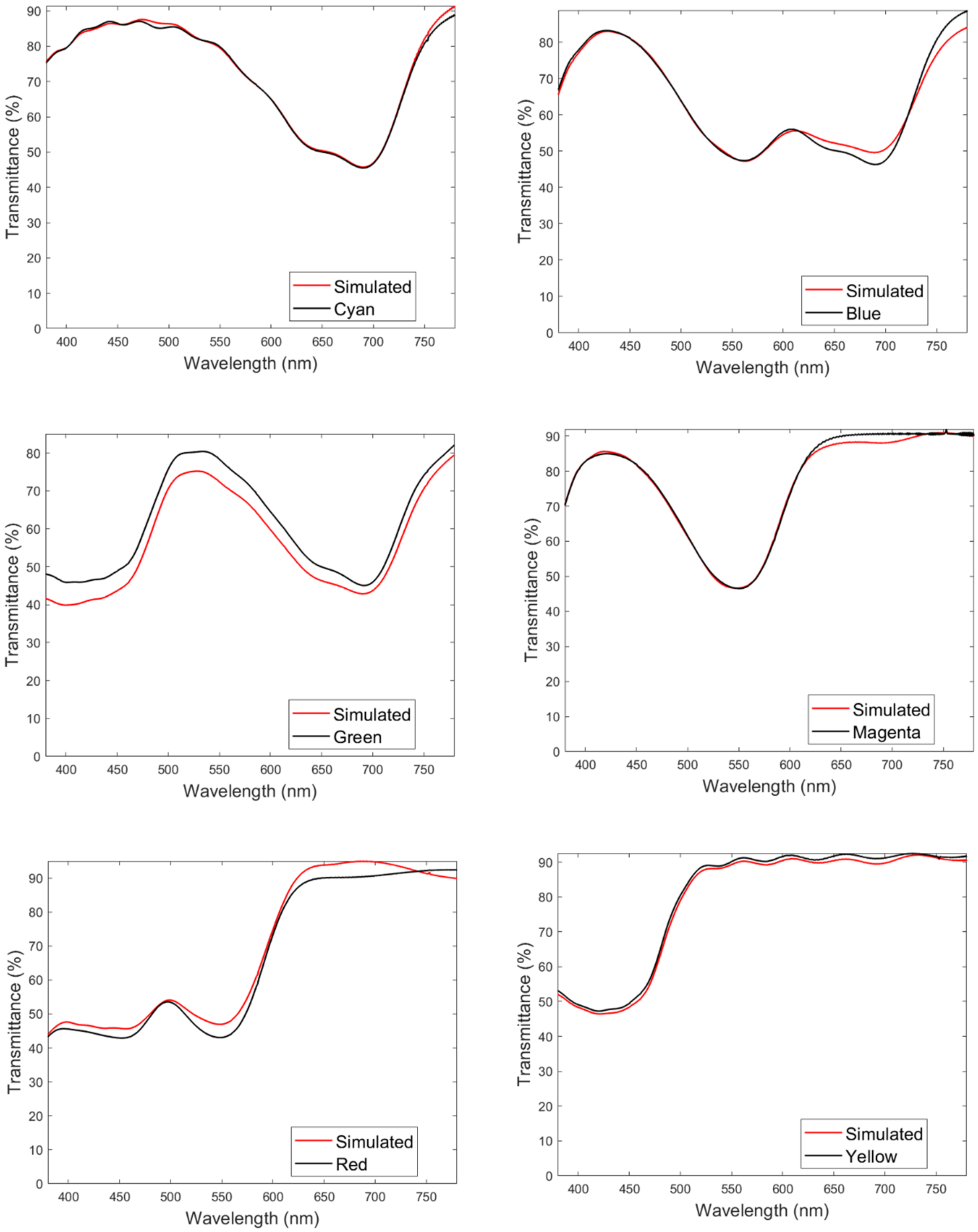

Figure S3. Original spectra (black curves) and simulated spectra (red curves) of color checkers for cyan, blue, green, magenta, red and yellow.

	Before					After			Color Dif.	
Color filters	L	a	b			L	a	b	CIE2000	CIE76
1	60.5626	-18.2909	55.6829			61.0158	-18.2957	55.1886	0.42475	0.67064
2	70.5415	-8.62676	59.6547			70.0981	-8.598	60.2701	0.3849	0.75896
3	79.0148	-35.7803	67.6608			78.6809	-36.0294	68.2788	0.27883	0.7453
4	42.4735	10.6805	35.8781			42.4529	10.7331	35.7806	0.06904	0.11266
5	43.7292	19.8369	-4.57738			47.4335	18.9918	-3.72042	3.61061	3.89492
6	71.261	-3.13569	31.0424			71.3598	-3.278	31.1852	0.14187	0.22455
7	58.4719	19.4852	13.2369			58.4157	19.4662	13.2945	0.06861	0.08268
8	69.275	-16.3116	27.2541			68.1669	-16.1894	26.9375	0.88207	1.15892
9	50.5743	23.7284	-20.719			48.5824	24.8819	-22.1459	2.13613	2.70817
10	62.6192	22.8625	-3.89154			62.7989	22.8931	-3.92816	0.15473	0.186
11	72.4724	-10.7947	10.2144			72.4203	-10.5098	9.59805	0.43151	0.68101
12	57.8811	-40.2179	51.5916			57.9414	-40.212	51.5641	0.05538	0.06664
13	68.3916	-23.2794	56.3806			68.9895	-22.9418	55.3994	0.54713	1.19758
14	60.4322	-31.775	16.9304			57.7242	-33.8555	16.2701	2.63577	3.47819
15	80.3803	-30.747	41.2022			82.2257	-30.6899	41.5136	1.26707	1.87229
16	71.2761	-12.2478	9.58745			73.4271	-12.9271	11.5037	2.03884	2.95979
17	78.956	-44.1771	38.4507			79.8741	-43.4563	38.2622	0.68229	1.18239
18	85.7041	-33.2693	44.7442			83.9891	-33.3907	44.5576	1.13444	1.72946
19	64.6482	-20.9981	-0.36659			64.9059	-20.8794	-0.2304	0.24374	0.31475
20	73.7783	-11.6948	10.9105			72.0247	-11.3322	9.80996	1.49597	2.10189
21	81.5813	-36.8807	21.5233			81.2658	-36.923	21.3065	0.24335	0.38514
22	26.7556	17.4003	-32.5381			29.8834	15.599	-29.3846	2.74353	4.793
23	49.0437	16.5068	-1.53347			46.9997	16.1632	-1.3799	2.03573	2.07839
24	62.3005	-28.0569	17.1618			63.5603	-27.7978	17.9211	1.16318	1.49353
									1.03623	1.4532

Figure S4. Color difference between simulated spectrum and measurement spectrum.

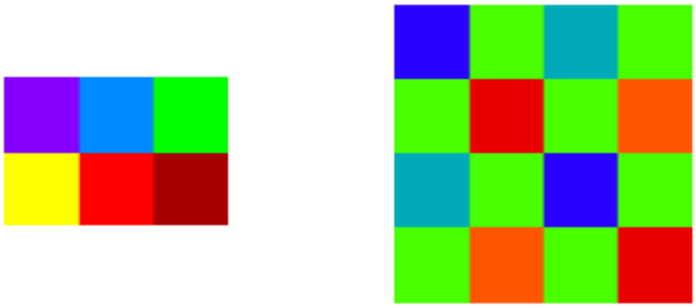

(a) (b)

Figure S5. Examples of MSFAs (a) Brauers and Aach [1] (b) Monno *et al.* [2].

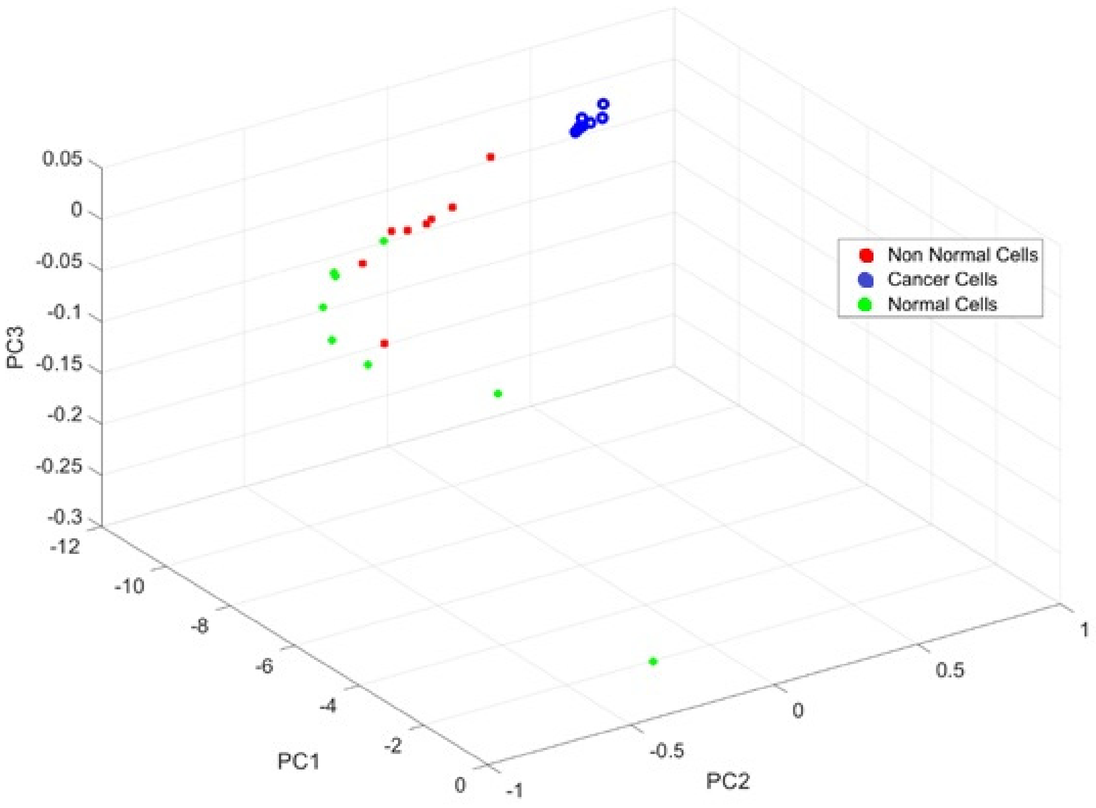

Figure S6. Principal component distribution diagram of the three kinds of cells

References

1. Brauers, J.; Aach, T. A color filter array based multispectral camera. In 12. Workshop Farbbildverarbeitung; Ilmenau, Germany, 2006.
2. Monno, Y.; Kikuchi, S.; Tanaka, M.; Okutomi, M. A practical one-shot multispectral imaging system using a single image sensor. *IEEE Trans. Image Process.* **2015**, *24*, 3048–3059. https://doi.org/10.1109/tip.2015.2436342.

Disclaimer/Publisher's Note: The statements, opinions and data contained in all publications are solely those of the individual author(s) and contributor(s) and not of MDPI and/or the editor(s). MDPI and/or the editor(s) disclaim responsibility for any injury to people or property resulting from any ideas, methods, instructions or products referred to in the content.
